# Infantile Systemic Hyalinosis: A Familiar Symptom Unveiling an Unusual Disease

**DOI:** 10.7759/cureus.103465

**Published:** 2026-02-12

**Authors:** Swastika Nayek, Arshpreet Sandhu, Amber Kumar, Shikha Malik

**Affiliations:** 1 Pediatrics, All India Institute of Medical Sciences (AIIMS) Bhopal, Bhopal, IND

**Keywords:** diffusely thickened skin, infantile systemic hyalinosis, intractable secretory diarrhea, protein-losing enteropathy, reduced joint mobility

## Abstract

Infantile systemic hyalinosis is a rare autosomal recessive disorder characterized by the widespread deposition of hyaline material in multiple organs leading to progressive multisystem involvement. Early clinical manifestations are often nonspecific and frequently result in diagnostic delay. We report a seven-month-old female infant born of a third-degree consanguineous marriage who presented with persistent watery diarrhea since early infancy with failure to thrive. Clinical examination showed coarse facial features, hyperpigmentation over joints, perianal rash, and markedly reduced joint mobility with preserved deep tendon reflexes. Laboratory evaluation showed anemia, neutrophilic leukocytosis, elevated inflammatory markers, hypoalbuminemia, and reduced immunoglobulin levels, while stool studies were non-contributory. Upper gastrointestinal endoscopy demonstrated scattered white mucosal lesions suggestive of lymphatic dilation or hyaline deposition. After the exclusion of infectious, metabolic, and malabsorptive causes, a genetic etiology was suspected. Whole exome sequencing identified a homozygous pathogenic frameshift mutation in the ANTXR2 gene (c.1074del; p.Ala359fs), confirming the diagnosis of infantile systemic hyalinosis. This case highlights the importance of considering rare genetic disorders in infants presenting with chronic diarrhea accompanied by joint contractures and skin lesions and emphasizes the role of early genetic testing for definitive diagnosis, appropriate counseling, and optimized supportive care.

## Introduction

Hyaline fibromatosis syndrome (HFS) is a rare autosomal recessive disorder resulting in the accumulation of hyaline material in the skin and various organs, characterized by skin lesions and joint contractures [1]. It is a unifying term for two related conditions: infantile systemic hyalinosis (severe form) and juvenile hyaline fibromatosis (mild form) [2]. Mutations in the capillary morphogenesis gene 2 (CMG2) are responsible for both conditions causing a lack of post-translational modification due to the retention of the protein interferon alpha-2b in the endoplasmic reticulum [3]. Prevalence is <1/1000000 worldwide [4]. Patients with a severe form of infantile systemic hyalinosis presented with intractable diarrhea leading to protein-losing enteropathy, failure to thrive, and early death [5].

Against this background, we reported a child with intractable diarrhea that was ultimately diagnosed as infantile systemic hyalinosis. The joint contractures and characteristic skin changes provided important clues towards the underlying genetic disorder.

As clinical features at onset overlap across a wide range of conditions, from common infections to connective tissue disorders to rare genetic syndromes, a systematic and stepwise approach is necessary to establish a proper diagnosis.

## Case presentation

A seven-month-old female child born to a third-degree consanguineous marriage was brought in with complaints of persistent loose stool for two months. It was watery, without any blood, non-foul-smelling, and of large volume, suggestive of secretory diarrhea. It was not associated with fever, vomiting, and decreased urine output with intact thirst and appetite. There was no history of similar complaints in the family. Anthropometric measurements were consistent with severe acute malnutrition.

**Figure 1:**
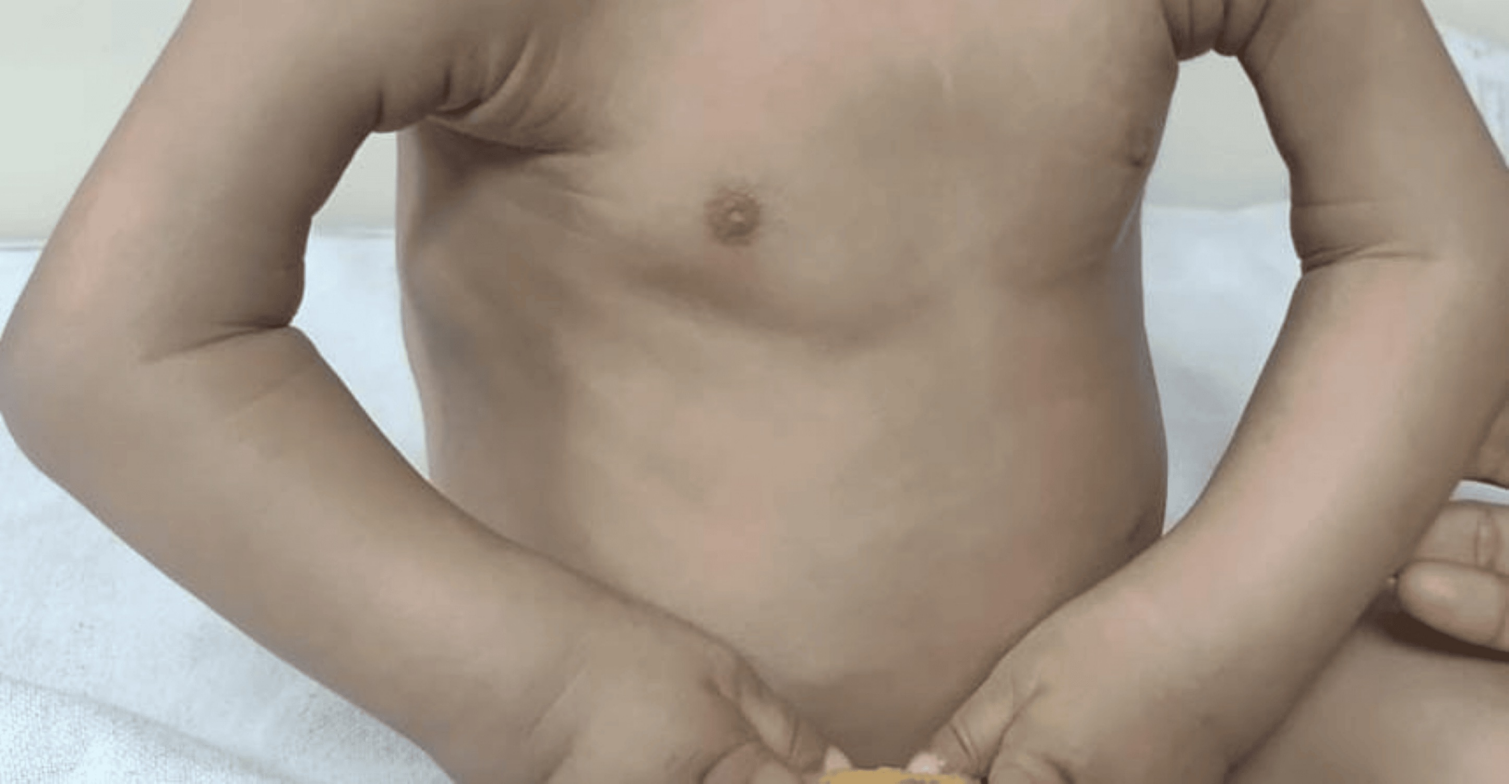
Joint contractures at bilateral elbow joints

**Figure 2:**
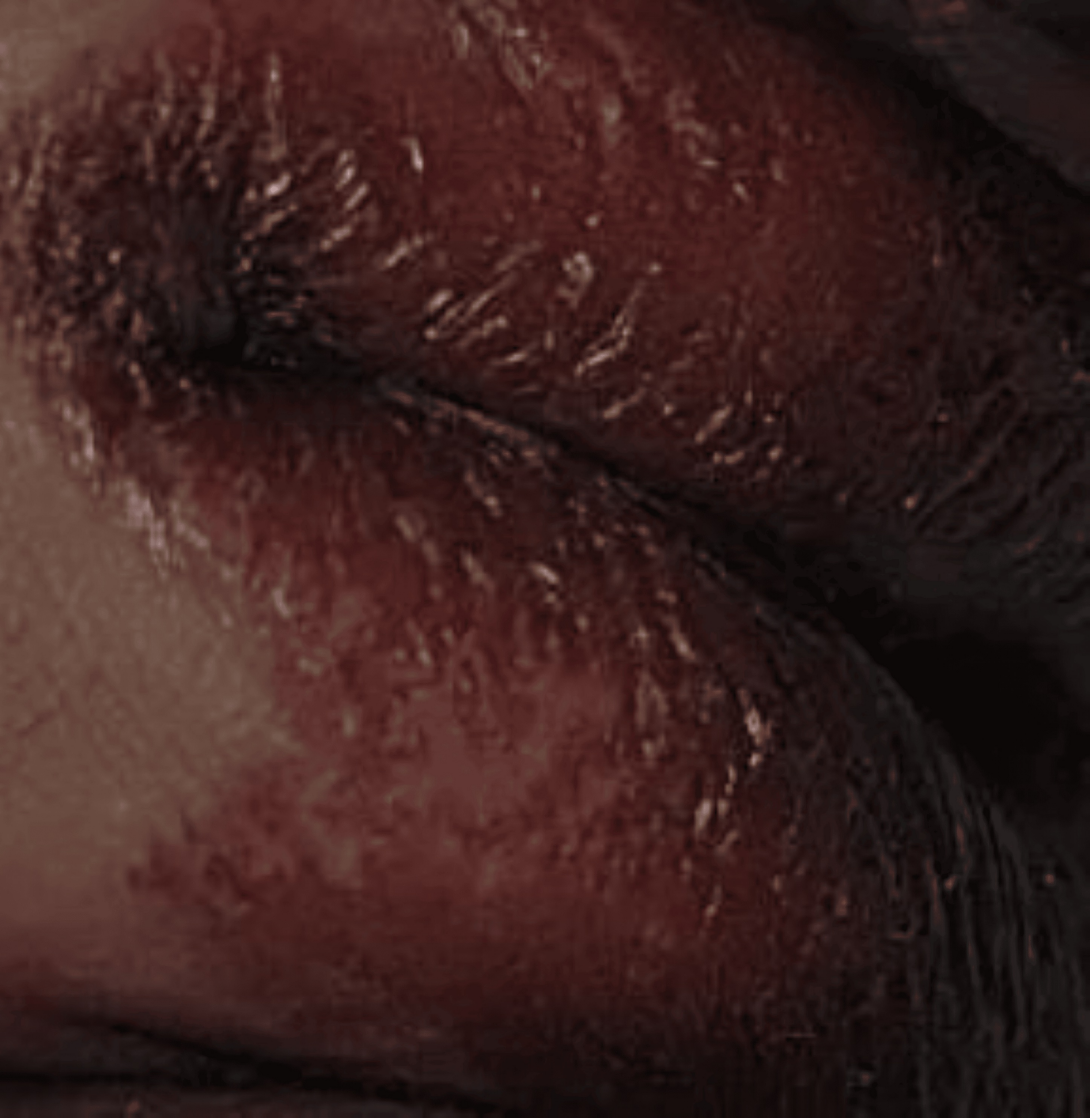
Erythematous thickened plaques over the perineal area

**Figure 3:**
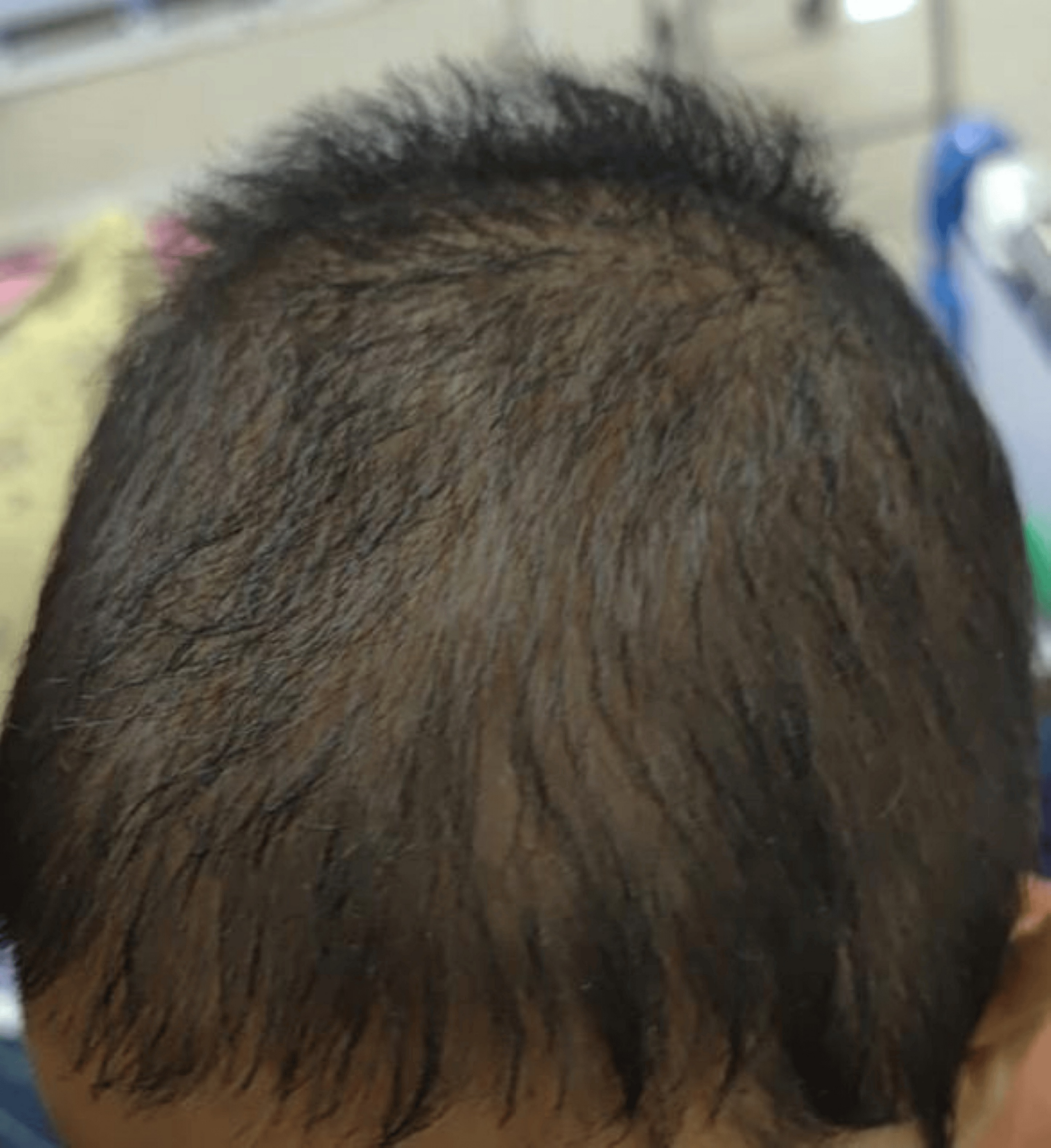
Short, coarse, spiky scalp hair with sparse distribution

**Figure 4:**
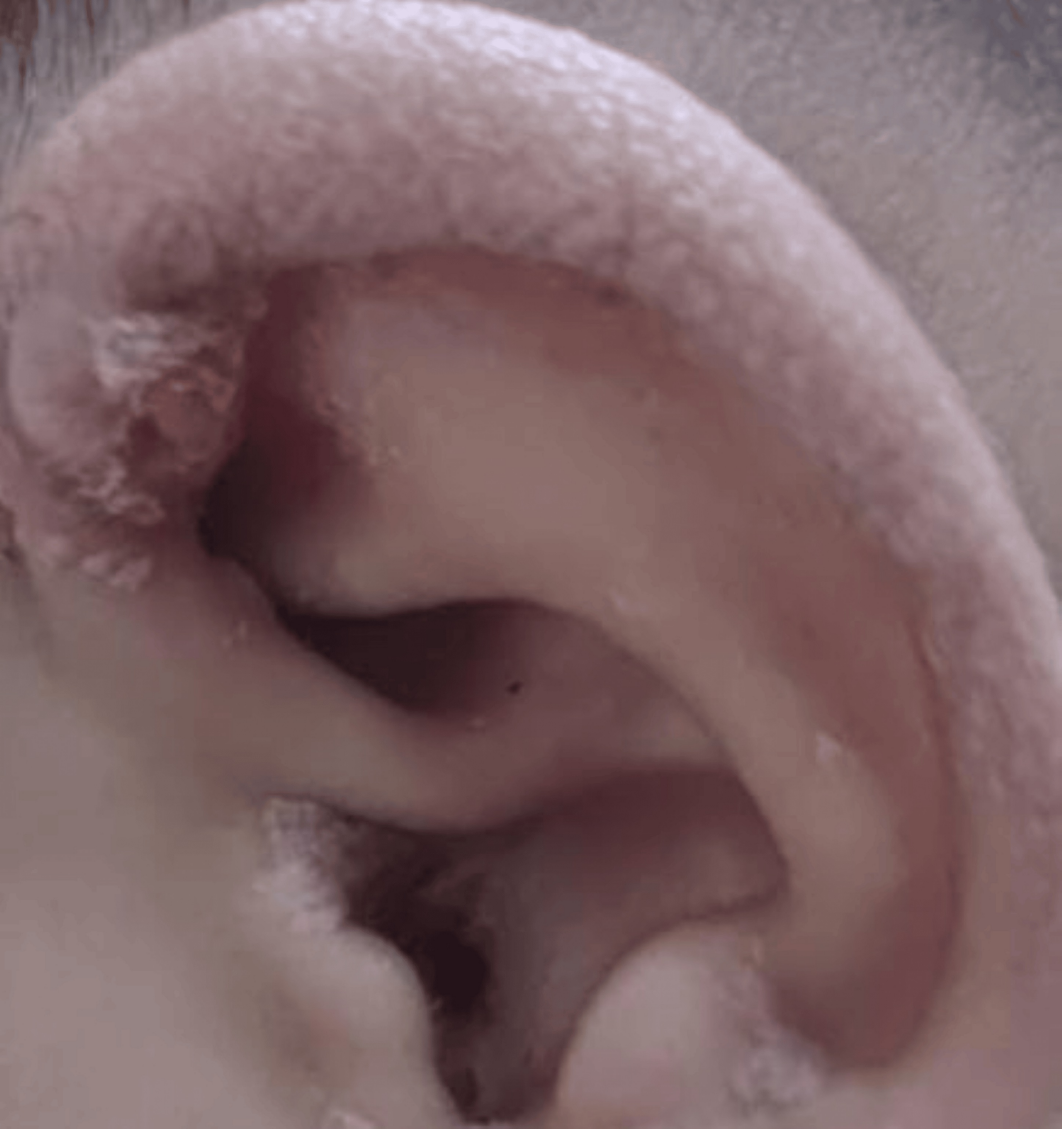
Nodular infiltrative lesion at the root of the helix with thickening of the pinna

**Figure 5:**
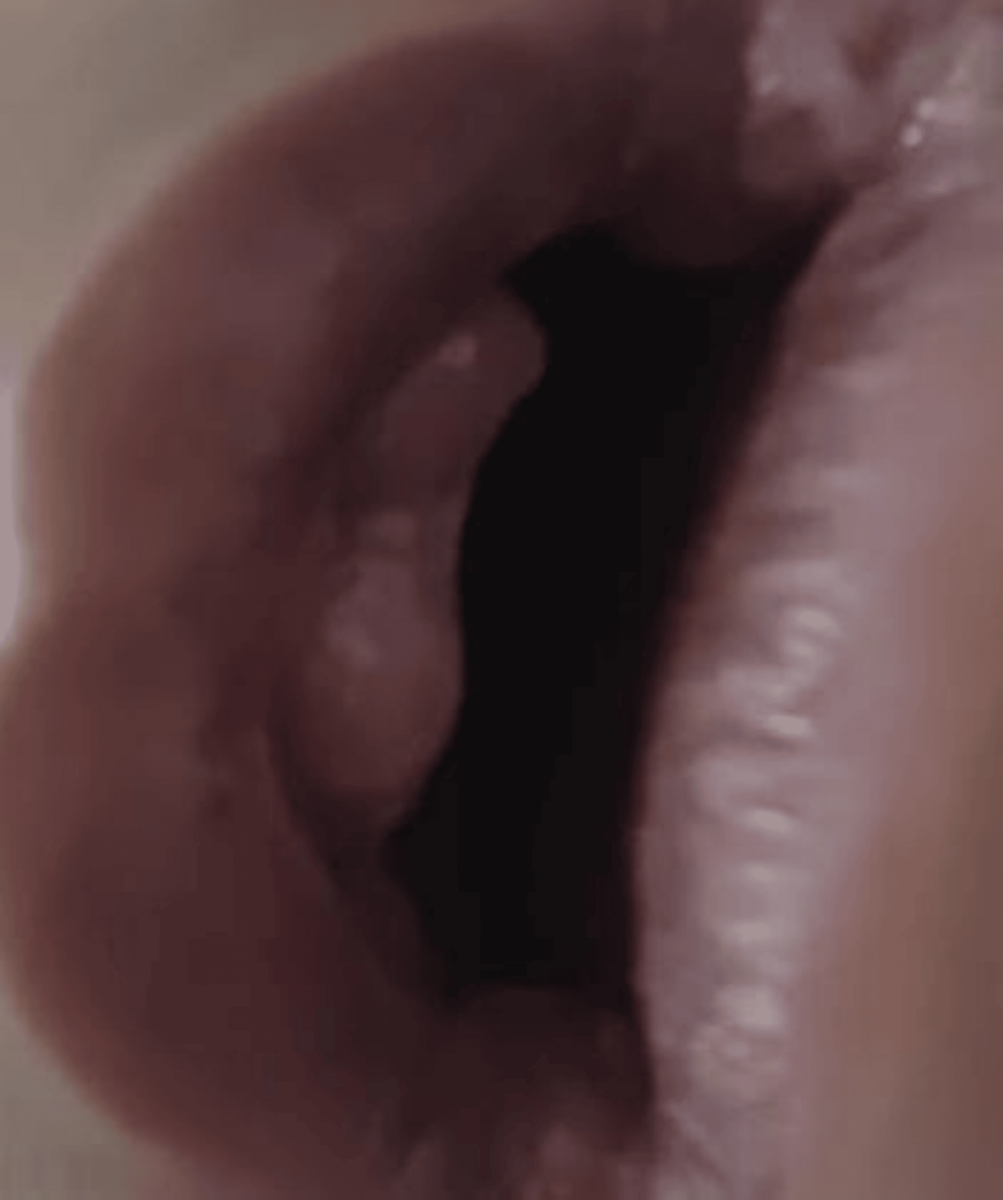
Gingival hypertrophy

On examination, the child appeared pale and had generalized anasarca. She had coarse facies with a broad nasal bridge and erythematous pinna with pink pearly papules on it along with preauricular skin tags and angular stomatitis. Her skin was diffusely thickened with hyperpigmentation over the metacarpophalangeal joints. She had bilateral elbow joint contractures (Figure 1). The mobility of the shoulder, elbow, wrist, hip, and ankle joints was strikingly diminished. There was diminished motor strength with intact deep tendon reflexes, which was considered a result of reduced joint mobility. A perineal erythematous thickened plaque was seen (Figure 2). She had short, straight, thick, spiky hair which was uniformly black in color and evenly distributed (Figure 3), a nodular infiltrative lesion at the root of the helix with thickening of the pinna (Figure 4), and joint gingival hypertrophy (Figure 5). No organomegaly was there.

Laboratory investigations revealed anemia, neutrophilic leukocytosis, elevated C-reactive protein (CRP), and hypoalbuminemia (Table 1). Stool routine and microscopic examination and ova and parasite test did not show any significant findings. The stool test was negative for fat globules and occult blood. Immunoglobulin was markedly reduced which was attributed to the protein-losing enteropathy.

**Table 1:** Laboratory parameters of the patient IgG: immunoglobulin G

Parameters	Patient values	Reference ranges
Hemoglobin	6.3 g/dL	11-15 g/dL
Total leukocyte count	18000/µL	4000-11000/µL
Urea	36 mg/dL	20-40 mg/dL
Creatinine	0.54 mg/dL	0.6-1.2 mg/dL
Aspartate aminotransferase	36.3 U/L	<50 U/L
Alanine aminotransferase	38.4 U/L	<50 U/L
Gamma-glutamyl transferase	50 U/L	<55 U/L
Albumin/globulin	1.14 g/dL	3.5-5.2 g/dL
Globulin	1.45 g/dL	1.9-3.7 g/dL
Total IgG	3.04 g/L	7-16 g/L
Total IgM	0.48 g/L	0.5-2 g/L
Total IgA	0.37 g/L	0.7-4 g/L

**Figure 6:**
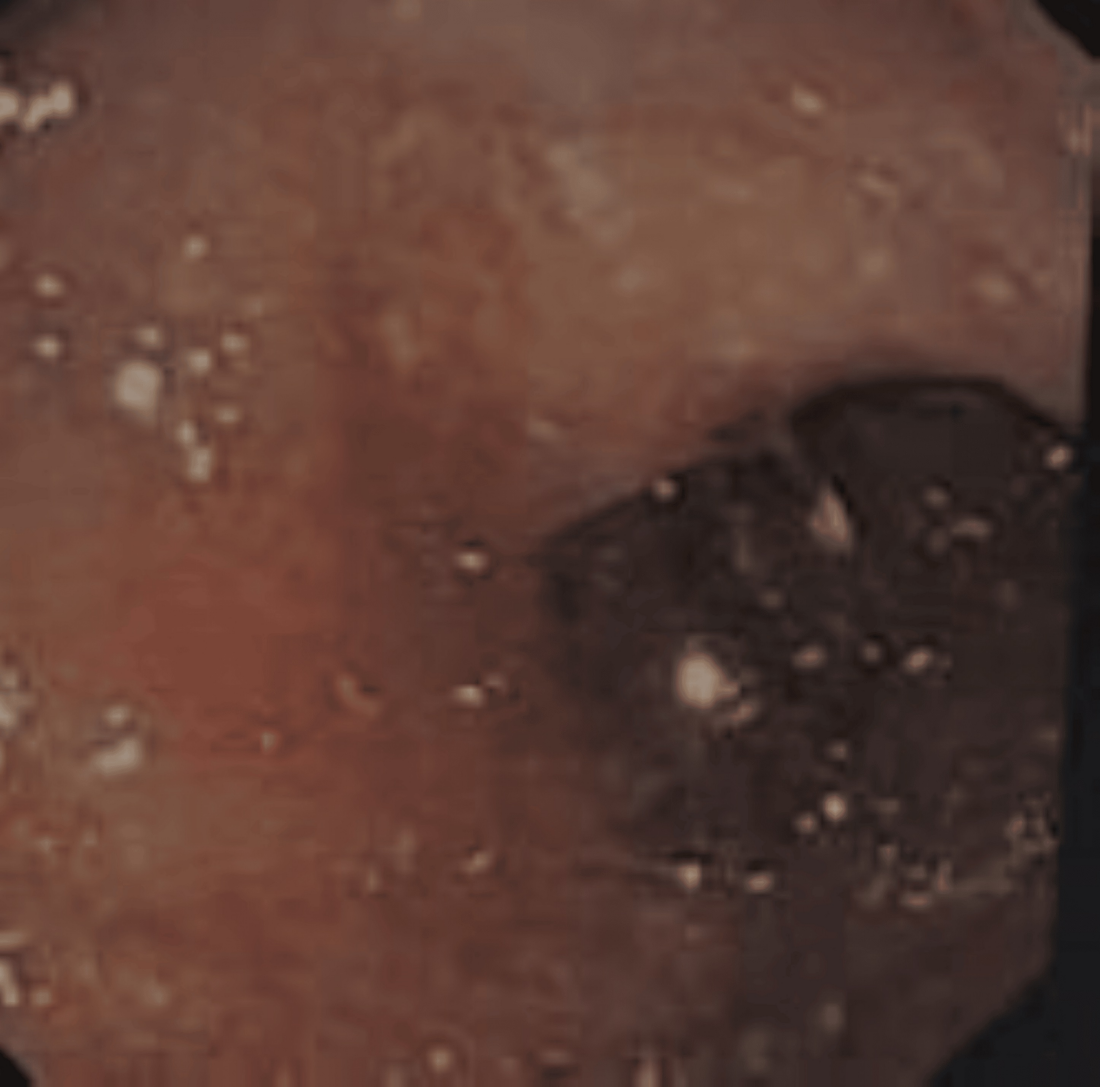
Scattered white spots in the duodenum during upper gastrointestinal endoscopy

The ultrasonography of the whole abdomen has no significant abnormality. Upper gastrointestinal endoscopy revealed a normal duodenal bulb, but the second part of the duodenum revealed multiple scattered white spots over the mucosal surface which can be suggestive of lymphatic dilation, mucosal edema, or hyaline deposition (Figure 6). Biopsy was not suggestive of any villous atrophy, dysplasia, or any dilated lymphatics.

Trichoscopy revealed no trichorrhexis nodosa and areas of thinning and hypopigmentation in between normal segments with normal width and pigmentation in the hair shaft. Skin biopsy could not be performed in view of parental refusal.

After ruling out common causes, attention shifted towards a genetic origin in view of joint and skin involvement and non-contributory biopsy findings.

Cystic fibrosis, congenital chloride diarrhea, and infantile systemic hyalinosis were kept in the differential diagnosis for intractable diarrhea.

However, blood gas was not suggestive of any metabolic alkalosis, hypochloremia, or hypokalemia. Stool electrolytes were within normal limits, which ruled out cystic fibrosis and congenital chloride diarrhea. Absence of recurrent respiratory tract infection and presence of joint contracture and skin lesions pointed towards infantile systemic hyalinosis.

Confirmation was done through whole exome sequencing which suggested a homozygous pathogenic frameshift variant in the ANTXR2 gene (c.1074del; p.Ala359fs50). The pathogenic mutation confirms the diagnosis of HFS.

Parents were counseled regarding the disease, treatment, prognosis, inheritance pattern, and recurrence risk in subsequent pregnancy. The patient was treated symptomatically with satisfactory nutrition with adequate calories and albumin infusion. She got symptomatically relieved and was reviewed at one month. Diarrhea was well controlled, and she had a stable weight and good acceptance of physiotherapy. However, improvement in motor function was minimal. She was kept on close follow-up.

## Discussion

HFS is a rare and progressive autosomal recessive genetic disorder resulting from loss-of-function mutations in the CMG2, also referred to as ANTXR2, a novel homozygous mutation [1].

The index child presented at seven months of age with intractable diarrhea, generalized edema, failure to thrive, joint stiffness, and characteristic skin changes.

The protein is involved in extracellular matrix homeostasis, collagen VI turnover, and endothelial cell integrity. Loss-of-function mutation leads to the massive accumulation of hyaline material leading to progressive organ dysfunction [6].

In addition to the skin, hyaline material accumulates in a wide range of tissues, including the dermis, gastrointestinal tract (intestines and stomach), skeletal muscles, myocardium, trachea, esophagus, lymphatic tissue (lymph nodes), adrenal glands, thyroid, spleen, and thymus. The hyaline material deposition stiffens periarticular connective tissue, leading to irreversible fibrosis and joint contractures [7].

In our case, the patient had also joint contractures and skin changes and infiltrative nodular lesions on her ears in view of hyaline material accumulation. She was managed conservatively by physiotherapy for joint contractures.

Abnormal hyaline material gets deposited in the lamina propria and submucosa layer of the small intestine, which disrupts normal tissue structure. These result in gland distension, epithelial flattening, and wall thickening which, respectively, cause crypt damage, surface area reduction, and mucosal atrophy. All of these collectively cause compromised digestion and absorption, resulting in intractable diarrhea [8].

This hyaline deposition compresses lymphatic channels, which prevents normal flow. Impaired lymph drainage causes lymphatic backflow, causing the dilation of mucosal lymphatics. Dilated lymphatic vessels become leaky and cause leaching into the intestinal lumen, resulting in protein-losing enteropathy [8]. There are various mechanisms of protein-losing enteropathy which can be due to mucosal erosion and ulceration.

Growth retardation is also a striking feature of this disease which can be explained by inadequate oral intake due to periorbital stiffness or excess protein loss from the intestine [8]. Our patient had severe acute malnutrition, and there was also no adequate weight gain on the follow-up visit.

Chronic diarrhea can mislead and delay diagnosis of this rare disease like infantile systemic hyalinosis, as clinical features are shared by many diseases. When diarrhea is accompanied by features such as joint contractures and characteristic skin lesions, a rare underlying systemic or genetic disorder should be suspected. This can be confirmed by genetic testing only.

Treatment is only supportive. Physiotherapy and nutritional rehabilitation improve quality of life. Remarkable improvement with interferon alpha-2b was reported, but confirmation is awaited.

The prognosis is very poor as most of the patients die within two years of life. Genetic counseling is needed before the next conception to inform the next child has a 25% chance of being affected.

## Conclusions

This case highlights how a rare disease manifests with common symptoms which lead to diagnostic delay. Here, whole exome sequencing played a definitive role in the diagnosis confirming the underlying mutation where clinical and biochemical laboratory investigations were inconclusive. Rare genetic disorders characterized by multisystemic manifestations need multidisciplinary management which is crucial to improve the quality of life of affected individuals as there is no permanent cure for these conditions. 
